# Establishment of a Dynamic Nomogram for Predicting the Risk of Lymph Node Metastasis in T1 Stage Colorectal Cancer

**DOI:** 10.3389/fsurg.2022.845666

**Published:** 2022-03-21

**Authors:** Zitao Liu, Chao Huang, Huakai Tian, Yu Liu, Yongshan Huang, Zhengming Zhu

**Affiliations:** Department of Gastrointestinal Surgery, The Second Affiliated Hospital of Nanchang University, Nanchang, China

**Keywords:** T1 stage colorectal cancer, lymph node metastasis (LNM), random forest, LASSO regression algorithm, dynamic nomogram

## Abstract

**Background:**

Accurate prediction of the risk of lymph node metastasis in patients with stage T1 colorectal cancer is crucial for the formulation of treatment plans for additional surgery and lymph node dissection after endoscopic resection. The purpose of this study was to establish a predictive model for evaluating the risk of LNM in patients with stage T1 colorectal cancer.

**Methods:**

The clinicopathological and imaging data of 179 patients with T1 stage colorectal cancer who underwent radical resection of colorectal cancer were collected. LASSO regression and a random forest algorithm were used to screen the important risk factors for LNM, and a multivariate logistic regression equation and dynamic nomogram were constructed. The C index, Calibration curve, and area under the ROC curve were used to evaluate the discriminant and prediction ability of the nomogram. The net reclassification index (NRI), comprehensive discriminant improvement index (IDI), and clinical decision curve (DCA) were compared with traditional ESMO criteria to evaluate the accuracy, net benefit, and clinical practicability of the model.

**Results:**

The probability of lymph node metastasis in patients with T1 colorectal cancer was 11.17% (20/179). Multivariate analysis showed that the independent risk factors for LNM in T1 colorectal cancer were submucosal invasion depth, histological grade, CEA, lymphovascular invasion, and imaging results. The dynamic nomogram model constructed with independent risk factors has good discrimination and prediction capabilities. The C index was 0.914, the corrected C index was 0.890, the area under the ROC curve was 0.914, and the accuracy, sensitivity, and specificity were 93.3, 80.0, and 91.8%, respectively. The NRI, IDI, and DCA show that this model is superior to the ESMO standard.

**Conclusion:**

This study establishes a dynamic nomogram that can effectively predict the risk of lymph node metastasis in patients with stage T1 colorectal cancer, which will provide certain help for the formulation of subsequent treatment plans for patients with stage T1 CRC after endoscopic resection.

## Introduction

Colorectal cancer is a common malignant cancer of the digestive tract and has the third-highest mortality rate among all cancers worldwide (1). In recent years, with the improvement of people's health awareness, the popularization of colorectal cancer screening programs and the improvement of endoscopic systems, many early colorectal cancers have been diagnosed and treated early (2, 3). At the same time, many T1 colorectal cancer patients who previously required radical surgical treatment can be cured by endoscopic mucosal resection (4). Previous studies have shown that the probability of lymph node metastasis in T1 colorectal patients is 7 to 16% (5–7). Therefore, accurate assessment of the risk of lymph node metastasis in patients with T1 colorectal cancer is an important factor in prognosis and whether additional surgical treatment is needed after endoscopic resection (8). At present, the assessment of LNM at stage T1 is mainly based on pathological specimens after endoscopic resection and related guidelines, such as those of the National Cancer Network (NCCN), European Society of Medical Oncology (ESMO), and Japanese Society of Colorectal Cancer (JSCCR). The risk factors for LNM are the depth of submucosal invasion (>1,000 μm), histological grade, vascular and lymphatic invasion, and tumor budding. Currently, there are some controversies regarding tumor budding as a risk factor for T1 stage colorectal cancer LNM, and only the JSCCR has included it in the guidelines (9–11). If patients have one or more risk factors, they are considered to be at high risk for LNM and require additional surgery and lymph node dissection. However, the way that patients are classified into low-risk and high-risk groups have led to a decrease in the positive predictive value of LNM and the result of overtreatment (12). According to the current guidelines, the incidence rate of LNM is ~10%, which means that many patients without lymph node metastasis have undergone unnecessary additional surgery, and there is a 1.5–3% probability of postoperative death. In addition, artificial anal surgery may be required for some patients with low stage T1 rectal cancer (13). Therefore, to reduce unnecessary surgery and provide suitable treatment for patients, it is necessary to establish a prediction model for accurately predicting LNM.

At present, although some studies have constructed scoring systems to stratify the risk of LNM for T1 colorectal cancer, Jung et al. constructed a nomogram prediction model that included five pathological factors (vascular invasion, submucosal invasion depth, tumor budding, differentiation grade, and adenoma background), with an AUC of 0.812 and a 95% CI of (0.770, 0.855) (6). Miyachi et al. constructed a prediction model that included five clinicopathological factors (mucosal muscular state, sex, vascular lymphatic invasion, differentiation grade, and tumor budding) (5). These models have the good discriminatory ability, but the risk factors included are mainly pathological factors, some of which are controversial. Recently, some studies have predicted the risk of LNM by observing the morphology and location of tumors and endoscopic features (ulcer depression, mucosal hypertonia, morphological changes, nodular eminence in the ulcer center, etc.) through endoscopic systems. However, because this study is a single-center study and endoscopic features such as LNM prediction factors are controversial, its clinical application is limited (7). There are also some studies that predict the risk of LNM through genomics and biomarkers. For example, Ozawa et al. found that high expression of five microRNAs (MIR32, MIR181B, MIR193B, MIR195, and MIR411) is closely related to lymph node metastasis of T1 colorectal cancer. The area under the ROC curve of this model is 0.83 (14). Kishida et al. found that the loss of ARID1A expression is related to LNM in T1 colorectal cancer (15). Kandimalla et al. showed the expression of 8 genes (AMT, MMP9, FOXA1, LYZ, MMP1, C2CD4A, PIGR, and RCC1) was related to LNM in T1 colorectal cancer. The area under the ROC curve of the prediction model was 0.88 (95% CI: 0.79–0.97) (16). Although these tumor markers can predict the risk of LNM relatively accurately, the complicated operation process limits their clinical application value.

Multi-slice spiral CT (MDCT) and MRI are routine imaging examinations for preoperative clinical evaluation of lymph node status in colorectal cancer patients. Lymph node metastasis is diagnosed through imaging features, including maximum short-axis diameter of lymph nodes, irregular morphology, necrosis of lymph node center, calcification of lymph nodes, the uneven density of lymph nodes, etc. (17–22). Although studies have shown that the accuracy of MDCT scanning in predicting LNM is 59–71% and that the accuracy, sensitivity, and specificity of MRI in predicting LNM are 92.5, 80–85, and 95%, respectively (17, 18, 23), there are few studies on T1 colorectal cancer. Therefore, this study constructs a prediction model based on the clinicopathological factors and imaging manifestations of patients to evaluate the risk of lymph node metastasis in stage T1 colorectal cancer and provides a reference for the formulation of subsequent treatment plans for stage T1 colorectal cancer patients after endoscopic resection.

## Methods

This research was approved by the Ethics Committee of the Second Affiliated Hospital of Nanchang University and implemented according to the Declaration of Helsinki Ethical Standards.

### Study Patients

This study included 179 patients with stage T1 colorectal cancer who underwent radical resection of colorectal cancer at the Gastrointestinal Surgery Department of the Second Affiliated Hospital of Nanchang University from January 2010 to October 2020, including 103 males and 76 females, with a median age 59.5 ± 11.8 years old, 59 cases of colon cancer, and 120 cases of rectal cancer. The inclusion criteria were as follows: 1. Patients with stage T1 colorectal cancer were treated by radical resection of colorectal cancer, and postoperative pathology was confirmed as stage T1 colorectal cancer. 2. No neoadjuvant radiotherapy or chemotherapy before surgery; 3. Patients without other tumors; and 4. Complete preoperative clinical data; Patients with familial polyposis, Lynch syndrome history, and ulcerative colitis were excluded.

Colorectal cancer patients underwent surgery according to CME or TME principles with intestinal resection and lymph node dissection. The surgical methods mainly included open surgery, laparoscopic-assisted radical colorectal cancer surgery, and complete laparoscopic radical colorectal cancer surgery.

### Assessment of Clinicopathological Factors

The clinicopathological characteristics of the patients were extracted from the hospital's electronic health record system, and retrospective data were collected, including sex, age, smoking history, tumor markers (CEA, CA199) and hemoglobin, neutrophil ratio, lymphocyte ratio, fibrinogen, and prealbumin values at the time of the patient's first admission. The location of the tumor was divided into the following: right colon (ileocecal, ascending colon, and liver flexure of colon), left colon (splenic flexure of the colon, descending colon, and sigmoid colon), and rectum. The size of the tumor was measured according to the maximum diameter of the tumor under endoscopy. The tumor morphology under endoscopy was divided into two types according to the Paris standards: polyp type and non-polyp type, where the polyp type includes the pedicle polyp type and non-pedicle polyp type, and the non-polyp type includes the flat raised type and depressed type (24). The depth of submucosal invasion was divided into SM1, SM2, and SM3 according to Kudo's standard (25). According to the histological classification of colorectal cancer by the World Health Organization, cancers were divided into low and high grades. Low-grade cancers include highly differentiated and moderately differentiated adenocarcinoma, while high-grade cancers include poorly differentiated adenocarcinoma, signet ring cell carcinoma, mucinous adenocarcinoma, and neuroendocrine carcinoma. Lymphovascular invasion is defined as the appearance of tumor cells in the stroma of microvessels or the lymphatic space. The background of adenoma was defined by the continuity of adenoma tissue and resected cancer tissue under a microscope.

### Interpretation of CT and MRI Images

All the patients with colon cancer underwent plain or enhanced CT scans of the whole abdomen before the operation, and all patients with rectal cancer underwent pelvic MRI and plain or enhanced CT scans of the whole abdomen before the operation. Patients ate a digestible juicy diet within 2 days before undergoing a plain CT scan or enhanced CT scan of the whole abdomen and took oral laxatives to clean the intestinal tract one day before the MRI examination. A CT scan was performed with a 64-slice multispiral CT scanner, with a slice thickness of 5 mm. MRI was performed with 3.0 T MRI, and T1WI, T2WI, and DWI were performed. All case images were independently evaluated by 2 experienced chief radiologists (specializing in abdominal CT or MRI), and the status of peri-intestinal lymph nodes was evaluated in combination with the endoscopy results. If the two results were inconsistent, they would be re-evaluated by another chief radiologist, and the results would be determined after comprehensive analysis. Whether lymph nodes appeared on the image was recorded, as was the maximum value of the short axis of the lymph nodes, though it was ignored when the size of the suspected lymph nodes was <3 mm because they are difficult to distinguish from vascular structures and non-specific soft tissue density. Patients with rectal cancer underwent MDCT and MRI examinations. If only one examination had positive imaging manifestations, the results of the positive imaging manifestations were recorded. If both examinations had positive imaging manifestations at the same time, the MRI results were the main images considered.

### Statistical Analysis

The normality of the data was analyzed by the single sample K–S test. If the measurement data were normally distributed, they were expressed as the mean ± SD; otherwise, they were described by the median and quartile range. Categorical variables were described by the ratio and 95% CI. In the univariate analysis, a *t*-test or Mann-Whitney *U* test was used for measurement data, and the chi-square test was used for categorical variables, LASSO regression, and the random forest algorithm were used to screen the risk factors for LNM, and then the important risk factors were included in the construction of the logistic regression equation. The best cut-off value of important risk factors was determined by ROC curve analysis. To visualize the analysis of the data, a dynamic nomogram was constructed to calculate the risk of LNM. The C index, corrected C index, area under the ROC curve, and calibration curve were used to evaluate the prediction ability of the model. The calibration curve was internally verified by the bootstrap method, and the net reclassification index (NRI), comprehensive discriminant improvement index (IDI), and clinical decision curve (DCA) were compared with the traditional ESMO standard to evaluate the accuracy, net benefit, and clinical practicability of the model.

All data were analyzed using SPSS 24.0 (SPSS Inc., Chicago, IL, USA) and R (version x64 4.0.3), including the rms, corrplot, glmnet, randomforest, rsconnect, DynNom, rmda, PredictABEL, and other toolboxes. *P* < 0.05 indicated the statistical significancew.

## Results

### Clinical Characteristics

The study showed that 20 patients had LNM (11.17%), including 14 males and 6 females, aged 58.5 ± 14.9 years. A total of 159 patients (88.83%) had no LNM, including 89 males and 70 females, aged 59.6 ± 11.4 years. There were 125 cases with negative lymph nodes on imaging, and 54 cases were found to have enlarged lymph nodes. The minimum value was 3 mm, the maximum value was 11 mm, and the best cut-off value was 5 mm. Patients with enlarged lymph nodes were divided into two groups: 0–5 and > 5 mm. In the patients with the LNM group, 3 cases had negative lymph nodes on imaging, 8 cases had lymph nodes 0–5 mm in size, and 9 cases had lymph nodes > 5 mm in size. In the patients without LNM, 122 cases had negative lymph nodes on imaging, 24 cases had lymph nodes 0–5 mm in size, and 13 cases had lymph nodes > 5 mm in size. Age (*P* = 0.889), sex (*P* = 0.233), smoking history (*P* = 0.543), tumor location (*P* = 0.141), hemoglobin (*P* = 0.486), fibrinogen (*P* = 0.699), preoperative CA199 level (*P* = 0.486), the ratio of neutrophils to lymphocytes (*P* = 0.350), and the ratio of fibrinogen to prealbumin (*P* = 0.325) were not associated with lymph node metastasis in stage T1 colorectal cancer (Table 1).

**Table 1:** Clinicopathological and imaging features of patients with stage T1 colorectal cancer.

**Factors**	**Total**	**LNM (–)**	**LNM (+)**	***P*-value**
Age (year)	59.5 ± 11.8	59.6 ± 11.4	58.5 ± 14.9	0.889
**Sex**				
Male	103 (57.5%)	89 (86.4%)	14 (13.6%)	0.233
Female	76 (42.5%)	70 (92.1%)	6 (7.9%)	
**Smoking**				
No	153 (85.5%)	135 (88.2%)	18 (11.8%)	0.543
Yes	26 (14.5%)	24 (92.3%)	2 (7.7%)	
**Tumor size (cm)**				
≤ 2	53 (29.6%)	51 (96.2%)	2 (3.8%)	0.042
>2	126 (70.4%)	108 (85.7%)	18 (14.3%)	
**Tumor location**				
Right colon	17 (9.5%)	17 (100%)	0 (0%)	0.141
Left colon	42 (23.5%)	38 (90.5%)	4 (9.5%)	
Rectum	120 (67.0%)	104 (86.7%)	16 (13.3%)	
**Background adenoma**				
No	44 (24.6%)	35 (79.5%)	9 (20.5%)	0.025
Yes	135 (75.4%)	124 (91.9%)	11 (8.1%)	
**Depth of submucosal invasion**				
Sm1	63 (35.2%)	62 (98.4%)	1 (1.6%)	0.002
Sm2	46 (25.7%)	42 (91.3%)	4 (8.7%)	
Sm3	70 (39.1%)	55 (78.6%)	15 (21.4%)	
**Histologic grade**				
Low	173 (96.6%)	158 (91.3%)	15 (8.7%)	<0.001
High	6 (3.4%)	1 (16.7%)	5 (83.3%)	
**Lymphovascular invasion**				
No	165 (92.2%)	150 (90.9%)	15 (9.1%)	0.002
Yes	14 (7.8%)	9 (64.3%)	5 (35.7%)	
**Tumor type**				
Polyp type	76 (42.5%)	72 (94.7%)	4 (5.3%)	0.032
Non-polyp type	103 (57.5%)	87 (84.5%)	16 (15.5%)	
**Imaging results**				
Node negative	125 (69.8%)	122 (97.6%)	3 (2.4%)	<0.001
Node size (0–5 mm)	32 (17,9%)	24 (75%)	8 (25%)	
Node size (>5 mm)	22 (12.3%)	13 (59.1%)	9 (40.9%)	
Hemoglobin (g/L)	125 ± 18.0	125.5 ± 17.6	120 ± 20.7	0.486
Fibrinogen (g/L)	2.83 ± 0.81	2.84 ± 0.82	2.72 ± 0.69	0.699
**Preoperative CEA (ng/ml)**				
≤ 5	165 (92.2%)	151 (91.5%)	14 (8.5%)	<0.001
>5	14 (7.8%)	8 (57.1%)	6 (42.9%)	
Preoperative CA199 (ng/ml)	13.54 ± 10.67	13.1 ± 10.26	16.8 ± 13.3	0.486
Neutrophil to lymphocyte ratio	2.69 ± 2.14	2.66 ± 2.20	2.98 ± 1.71	0.350
Fibrinogen to prealbumin ratio	0.01 ± 0.007	0.01 ± 0.007	0.01 ± 0.005	0.325

### Univariate and Multivariate Analysis

Univariate analysis showed that tumor size (*P* = 0.042), adenoma background (*P* = 0.025), submucosal invasion depth (*P* = 0.002), histological grade (*P* < 0.001), vascular and lymphatic invasion (*P* = 0.002), tumor type (*P* = 0.032), imaging results (*P* < 0.001) and preoperative CEA (*P* < 0.001) were related to lymph node metastasis in T1 colorectal cancer (Table 1). Multivariate analysis showed that submucosal invasion depth (OR =14. 997, *P* = 0.043), histological grade (OR = 42. 071, *P* = 0.006), preoperative CEA (OR = 5. 60, *P* = 0.028), vascular and lymphatic invasion (OR = 23. 891, *P* = 0.002), lymph node size 0–5 mm (OR = 5. 284, *P* = 0.049) and lymph node size > 5 mm (OR = 19. 074, *P* = 0.001) were independent risk factors for lymph node metastasis in T1 colorectal cancer (Table 2).

**Table 2:** Multivariate analysis of risk factors for lymph node metastasis in T1 colorectal cancer.

**Risk factors**	**B**	**SE**	**Wals**	***P*-value**	**OR (95%CI)**
**Depth of submucosal invasion**					
Sm1	1 (reference)				
Sm2 or Sm3	2.708	1.336	4.11	0.043	14.997 (1.094, 205.592)
**Histologic grade**					
Low	1 (reference)				
High	3.739	1.354	7.624	0.006	42.071 (2.959, 598.106)
**CEA (ng/ml)**					
≤ 5	1 (reference)				
>5	1.723	0.786	4.805	0.028	5.60 (1.20, 26.134)
**Imaging results**					
Node negative	1 (reference)				
Node size (0–5 mm)	1.665	0.847	3.867	0.049	5.284 (1.005, 27.766)
Node size (>5 mm)	2.948	0.847	12.11	0.001	19.074 (3.625, 100.374)
**Lymphovascular invasion**					
No	1 (reference)				
Yes	3.174	1.031	9.483	0.002	23.891 (3.17, 180.073)

### LASSO Regression and Random Forest Algorithm

**Figure 1:**
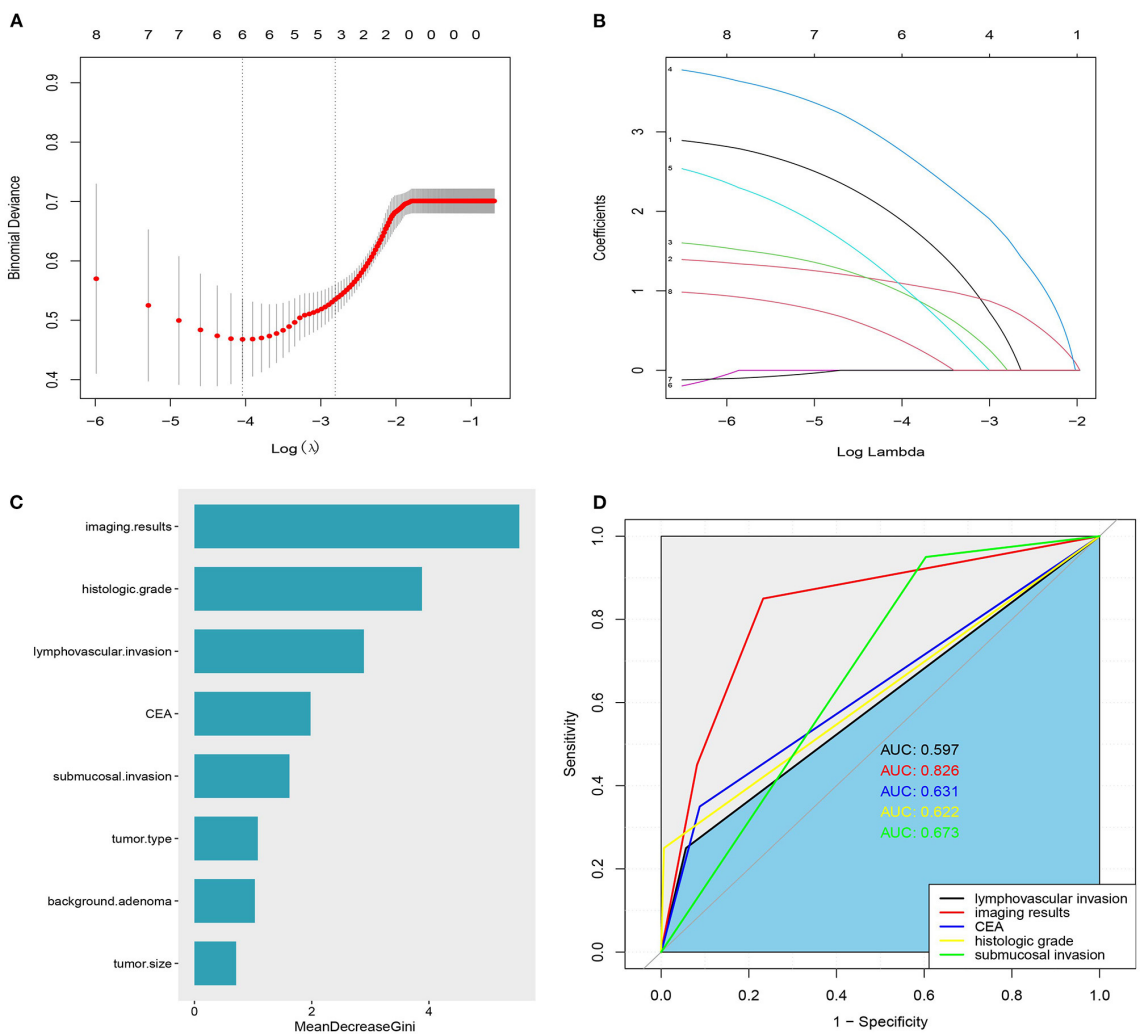
**(A)** Variable selection using the least absolute shrinkage and selection operator (LASSO) regression model. **(B)** Lasso coefficients were shown for 8 variables: 1: Lymphovascular invasion, 2: imaging results, 3: CEA,4: Histologic grade, 5: depth of submucosal invasion, 6: tumor size, 7: adenoma background, and 8: tumor type. **(C)** The importance of LNM related factors in T1 colorectal cancer was ranked. **(D)** ROC curve of independent risk factors of LNM in T1 colorectal cancer.

To avoid the influence of confounding factors, 8 significant factors from the univariate analysis were included in the LASSO regression, and variables were re-evaluated. Finally, 6 non-zero coefficient variables were included in the multivariate analysis. These factors were vascular lymphatic invasion, histological grade, imaging results, preoperative CEA, submucosal invasion depth, and tumor type (Figures 1A,B). In addition, the random forest algorithm was used to analyze the 8 factors that were of great significance in the single factor analysis, and the importance of the variables was ranked. The greater the Gini coefficient, the greater the importance of the factor. The imaging results ranked first, followed by histological grade, vascular and lymphatic invasion, preoperative CEA, and submucosal invasion depth (Figure 1C).

### Receiver Operating Characteristic Curve

Figure 1D shows the area under the curve of independent risk factors for lymph node metastasis in T1 colorectal cancer. According to ROC curve analysis, the cut-off value of the maximum diameter of the short axis of the enlarged lymph node on imaging was 5 mm. The area under the curve of vascular and lymphatic invasion was 0.597, 95% CI (0.451, 0.742), the sensitivity was 25%, and the specificity was 94.3. The area under the curve of the histological grade was 0.622, 95% CI (0.473, 0.771), the sensitivity was 25%, and the specificity was 99%. The area under the curve of the depth of submucosal invasion was 0.673, 95% CI (0.570, 0.776), the sensitivity was 95%, and the specificity was 39.6%. The area under the curve of preoperative CEA was 0.631, 95% CI (0.478, 0.772), the sensitivity was 30%, and the specificity was 95%. The area under the curve of the imaging results was 0.826, 95% CI (0.726, 0.926), the sensitivity was 85%, and the specificity was 76.6%. The results showed that only the imaging results had good sensitivity and specificity in predicting LNM, while the sensitivity or specificity of other factors was relatively low (Table 3).

**Table 3:** Independent risk factors for LNM in T1 colorectal cancer of ROC curve.

**Factors**	**AUC**	**95%CI**	**Sensitivity (%)**	**Specificity (%)**	**Cut-off value**
Lymphovascular invasion	0.597	(0.451, 0.742)	25.0	94.3	
Histologic grade	0.622	(0.473, 0.771)	25.0	99.0	
submucosal invasion	0.673	(0.570, 0.776)	95.0	39.6	
CEA	0.631	(0.478, 0.772)	30.0	95.0	
Imaging results	0.826	(0.726, 0.926)	85.0	76.6	5 mm

### Nomogram Construction

**Figure 2:**
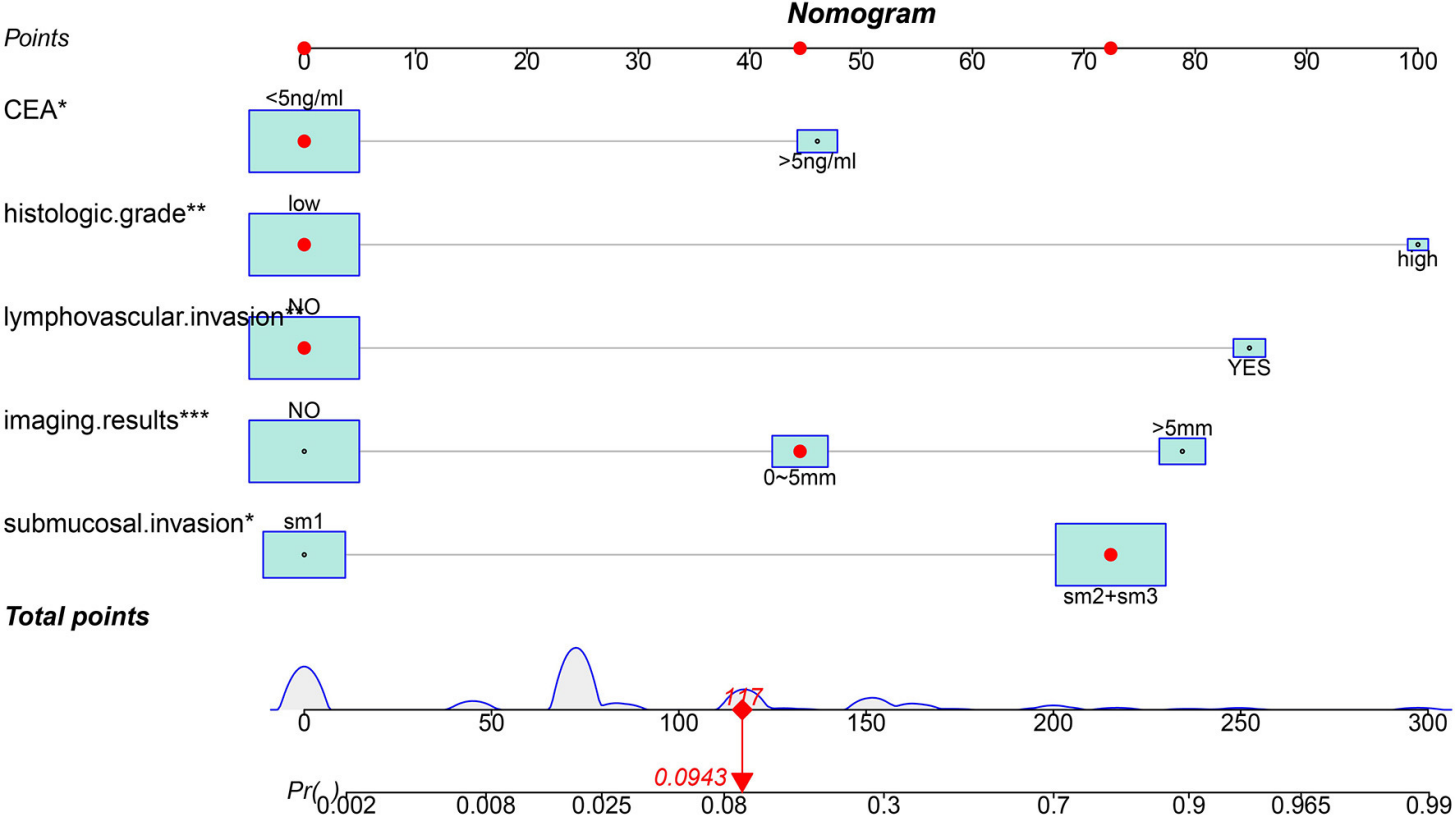
The nomogram for predicting lymph node metastasis in T1 colorectal cancer.

**Figure 3:**
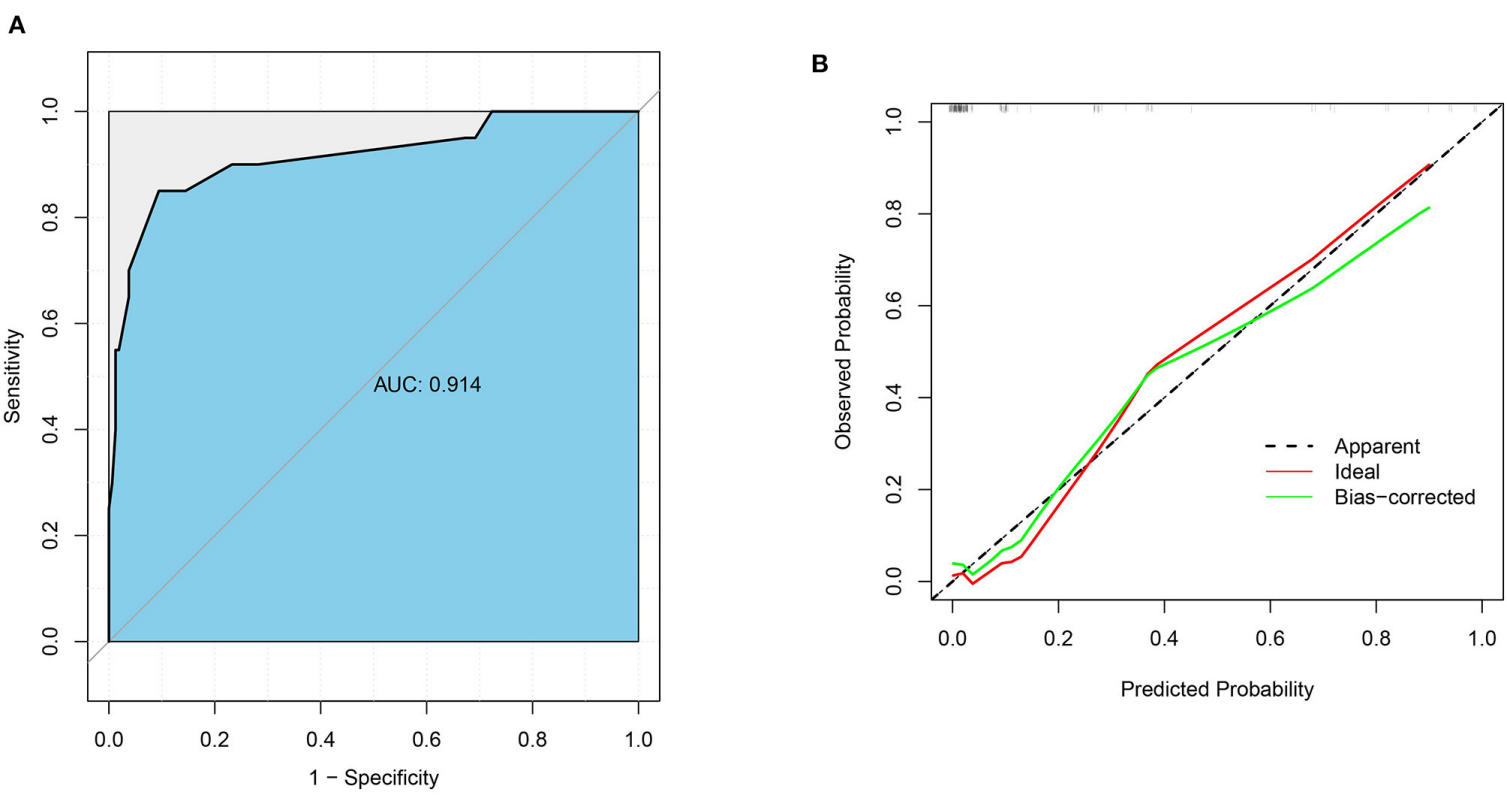
**(A)** ROC Curve Area of Prediction Model. **(B)** Calibration Curve of Nomogram Model.

Based on the results of logistic analysis, *R* was used to construct a prediction model (Figure 2), and each risk factor was assigned a value. The score of no vascular lymphatic invasion was 0, and the score of accompanying vascular lymphatic invasion was 85. The preoperative CEA ≤ 5 ng/ml score was 0, and the preoperative CEA > 5 ng/ml score was 46; 0 points indicated low-grade cancer, and 100 points indicated high-grade cancer. The submucosal invasion depth sm1 was 0, and sm2 or sm3 was 72. On imaging, the negative lymph nodes were 0 points, and the enlarged lymph nodes were 45 points for 0–5 mm and 79 points for > 5 mm. The total score of the prediction model was 382, suggesting that the risk of lymph node metastasis in T1 colorectal cancer was > 99%, and the risk of LNM was predicted by the sum of the scores (Table 4). The C index of the model was 0.914, the corrected C index was 0.890, the accuracy, sensitivity, and specificity were 93.3, 80, 91.8%, respectively, and the area under the ROC curve (Table 5) was 0.914 (Figure 3A). These results reflect that the prediction model has better discrimination ability and higher accuracy. The calibration curve also shows that the prediction model is highly consistent with the actual situation (Figure 3B). In addition, for the convenience of clinicians, we constructed a dynamic nomogram (https://liuzitao.shinyapps.io/dynnomapp/). When using this program, we only need to input the information for five patient variables, and we can immediately obtain the risk probability of LNM and the 95% CI.

**Table 4:** The relationship between total points and risk of LNM in T1 colorectal cancer.

**Total points**	**Risk of LNM**
<100	<5%
101–119	6–10%
120–140	11–20%
141–155	21–30%
156–167	31–40%
168–177	41–50%
178–188	51–60%
189–200	61–70%
201–214	71–80%
215–236	81–90%
237–256	91–95%
257–300	95–99%
>300	>99%

**Table 5:** Comparison of three model capabilities.

	**AUC**	**Sensitivity**	**Specificity**	**NRI**	**95%CI (%)**	**IDI**	**95%CI (%)**	**IDI *P*-value**
Model1	0.914	80.0%	91.8%	21.8%	(−2.95, 46.59)	12.58%	(1.85, 23.31)	0.021
Model2	0.850	65.0%	96.2%	13.7%	(−4.22, 31.64)	4.05%	(−0.83, 8.93)	0.104
Model3	0.817	50.0%	98.7%	reference	reference	reference	reference	reference

**Figure 4:**
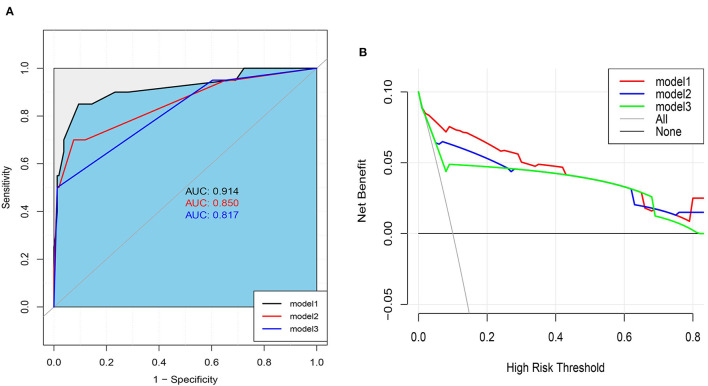
**(A)** Area under ROC Curve of Three Models. **(B)** Decision Curve analysis for Nomogram and ESMO Guidelines (model 3) and model 2 in Predicting LNM Risk of T1 Colorectal Cancer.

According to the ESMO guidelines, we constructed model 3 (composed of vascular lymphatic invasion, submucosal invasion depth, and histological grade) and added variable factors for preoperative CEA on the basis of model 3 to construct model 2. Compared with the ESMO guideline standard (model 3), the NRI of our prediction model (model 1) and model 2 were 21.8, 95% CI (−2.95,46.59), and 13.7, 95% CI (−4.22, 31.64), respectively, and the comprehensive discrimination improvement index (IDI) was 12.58, 95% CI (1.85, 23.31), *P* = 0.021, and 4.05, 95% CI (−0.83, 8.93), *P* = 0.104, respectively (Table 5, Figure 4A). These results show that our predictive model (model 1) is significantly superior to the ESMO guideline standard (model 3) and model 2 in predicting LNM.

A clinical decision curve (DCA) was drawn to compare the clinical utility and net benefit of our prediction model with ESMO guidelines (Model 3) and Model 2 (Figure 4B). The DCA curve shows that our prediction model has better clinical practicability than the other two models and obtains greater net benefits.

## Discussion

At present, there is some controversy about whether additional surgical treatment is needed for patients with stage T1 colorectal cancer after endoscopic resection and negative margins. Previous studies have shown that in stage T1 colorectal cancer, the 5-year overall survival rate (OS) of patients without lymph node metastasis is significantly higher than that of patients with lymph node metastasis (26). Therefore, accurate assessment of lymph node status in T1 colorectal cancer patients after endoscopic resection is an important factor in the formulation of treatment strategy and prognosis. Relevant guidelines such as the National Cancer Network (NCCN), the European Society of Medical Oncology (ESMO), and the Japanese Society of Colorectal Cancer (JSCCR) indicate that pathological factors such as vascular lymphatic invasion, histological grade, depth of submucosal invasion, and tumor budding are closely related to lymph node metastasis, and the aforementioned pathological factors are taken as indications for additional surgery after endoscopic resection (9–11). The multivariate analysis in this study also showed that vascular lymphatic invasion, histological grade, and depth of submucosal invasion were independent risk factors for lymph node metastasis in T1 colorectal cancer. In addition, preoperative CEA level and imaging results were also independent risk factors for LNM. Since tumor budding was not included in this study, comparing our prediction model with ESMO guidelines, it was found that the prediction model of this study has significantly better discrimination ability, accuracy, and clinical practicability for LNM than ESMO guidelines.

There is no doubt that vascular lymphatic invasion and differentiation grade are important factors in predicting LNM. Ichimasa et al. predicted the risk of LNM in T1 colorectal cancer through artificial intelligence analysis. The results showed that the importance of vascular lymphatic invasion and histological grade in the model was significantly greater than that of other clinicopathological factors (27). Our research also confirms this result. The cause of lymph node metastasis in high-histological grade tumors is generally related to a poor degree of differentiation in tumor cells, such as poorly differentiated adenocarcinoma, signet ring cell carcinoma, mucinous adenocarcinoma, and neuroendocrine carcinoma. These tumor cells have stronger invasion ability and are more likely to invade surrounding tissues, especially lymphatic tissues (28). In addition, vascular lymphatic invasion and high histological grade are also closely related to prognosis (26).

In this study, the depth of submucosal invasion was measured according to the Kudo criteria (sm1, sm2, and sm3). Multivariate analysis showed that the depth of submucosal invasion was also an independent risk factor for LNM, but compared with other clinicopathological features, the depth of submucosal invasion was the least important. According to the guidelines of the Japan Colorectal Cancer Society in 2019, when the submucosal invasion depth is <1,000 μm or close to 1,000 μm, the probability of lymph node metastasis is extremely low, at 1.3% (95% CI is 0–2.4%), and there is even no possibility of lymph node metastasis (10). Kitajima et al. also showed that a submucosal invasion depth <1,000 μm, a probability of LNM of 0, and a submucosal invasion depth >1,000 μm were risk factors for lymph node metastasis (29). Recently, some studies have suggested that a submucosal invasion depth >1,000 μm is a risk factor for LNM. Some controversy exists. Miyachi et al. reported that LNM occurred in 5 out of 61 lesions with submucosal invasion depths <1,000 μm (8.2%) (5), and Suh et al. also found that LNM occurred in 12 out of 98 SM1 lesions (12.2%) (30). In this study, it was also found that one case (1.6%) of SM1 had LNM. In Ei Kudo et al. and other studies, the risk of LNM for stage T1 colorectal cancer was analyzed through big data artificial intelligence algorithms. The results showed that the discrimination ability of the model that did not include tumor budding and submucosal invasion depth as predictive factors was better than that of the Japanese Colorectal Cancer Society (JSCCR) guidelines (31). In addition, the measurement of submucosal invasion depth has certain problems in the observation consensus of different pathologists, and different tumor types and diagnostic techniques will also affect the final results (32, 33). Therefore, the depth of submucosal invasion as a risk factor for LNM needs further study.

Most colorectal cancers evolve from adenomatous polyps, but ~30% of cancers appear directly in the form of cancer nests without adenoma evolution (34–36).^.^Previous studies have shown that a lack of adenoma background is closely related to LNM in T1 colorectal cancer (30). Studies by Ryul Oh et al. have shown that a lack of adenoma background is an independent risk factor for LNM (6), but studies by Miyachi et al. and Ichimasa et al. have shown that a lack of adenoma background is not significantly related to LNM (5, 27). In this study, univariate analysis showed that a lack of adenoma background was significantly associated with LNM, but multivariate analysis showed that a lack of adenoma background was not an independent risk factor for LNM. Recently, some studies have shown that clinicopathological features such as age, sex, tumor location, and tumor size are also related to LNM. For example, the risk of LNM for rectal cancer is higher than that for colon cancer (7, 37), the probability of LNM for women is significantly higher than that for men (5, 38, 39), and the risk of LNM for young patients is higher than that for patients of other ages (38, 40). However, as predictors of LNM, these clinicopathological factors are still debatable, and more research is needed to further confirm their predictive ability.

Many studies have confirmed that tumor markers have certain clinical value in evaluating lymph node metastasis, recurrence, distant metastasis, and prognosis of colorectal cancer (41, 42). However, there are some controversies about the clinical value of tumor markers in LNM evaluation of T1 colorectal cancer. Sun et al's research found that an increase in preoperative CA724 levels is a good predictor of LNM in T1 colorectal cancer (26). Mo et al.'s research showed that there is a certain correlation between preoperative CEA level and LNM (43). Guo et al.'s research was based on the SEER database and analyzed the risk factors for LNM in T1 colorectal cancer; it confirmed that the preoperative CEA level is related to LNM (38). In our study, we also found that an increase in the preoperative CEA level was an independent risk factor for LNM, but there was no obvious correlation between the preoperative CA199 level and LNM. Therefore, future research on tumor markers will help us to further understand the risk of LNM in T1 colorectal cancer.

The National Cancer Network (NCCN) recommends enhanced CT scans and MRI as effective imaging examinations for evaluating preoperative lymph node status of advanced colorectal cancer (9, 44). Radiologists evaluate lymph node status through imaging features, including the maximum diameter of the short axis of the lymph node, shape, lymph node density, and calcification (17–22). However, many criteria are not suitable for the preoperative assessment of lymph node status in T1 colorectal cancer. Previous studies indicated that if the maximum diameter of the short axis of lymph nodes on CT images is >1 cm, it is indicative of the metastatic lymph nodes (17, 45). If the maximum diameter of the short axis of lymph nodes on MRI images is >8 mm, the shape is irregular, the boundary unclear and other characteristics, it is indicative of metastatic lymph nodes (46). With the continuous improvement of CT and MRI equipment, lymph nodes as small as 5 mm can be detected on CT and MRI. In addition, pathologists have found that many lymph nodes smaller than 5 mm have metastasis. Therefore, an optimal cut-off value of lymph node size is very important for preoperative evaluation of lymph node status in T1 colorectal cancer. Choi et al. showed that the best cut-off value of the maximum diameter of the lymph node short axis was 4.1 mm, and the sensitivity and specificity were 78.6 and 75%, respectively (47). The best cut-off value in Kitaguchi et al. was 4.0 mm, and the sensitivity, specificity, accuracy, and negative predictive value of CT were 84, 69, 71, and 97%, respectively. The sensitivity, specificity, accuracy, and negative predictive value of MRI were 94, 23, 33, and 95%, respectively (48). The best cut-off value in this study was 5.0 mm. According to the cut-off value, the patients were divided into the imaging-negative lymph node group, the 0–5 mm group, and the > 5 mm group. The sensitivity and specificity were 85.0 and 76.6%, respectively, and the AUC was 0.826. These results show that a cut-off value of 5 mm is the standard and has a better ability to distinguish the status of lymph nodes before surgery. Therefore, preoperative combined imaging examination is helpful for accurately evaluating the status of lymph nodes before surgery. However, because of the lack of MRI examinations in colon cancer patients in this study, it is impossible to distinguish the diagnostic efficacy of CT and MRI for LNM in T1 colorectal cancer.

There are some limitations to this study. First, this study is a single-center retrospective study, and the sample size is relatively small, so there may be some selection bias. Therefore, the results need further verification with multicenter, big data research. Second, our prediction model has not been externally verified, which limits its value for clinical use to a certain extent. Third, all the patients in the study underwent radical surgery. Therefore, there may be some errors in the application of these risk factors to evaluate the lymph node status of patients after endoscopic resection, and further research is required.

## Conclusion

The prediction model of this study can accurately predict the risk of T1 stage colorectal lymph node metastasis, provide some help for whether patients with T1 stage colorectal cancer need additional surgical treatment after endoscopic resection, and help to reduce unnecessary operations.

## Data Availability Statement

The original contributions presented in the study are included in the article/Supplementary Material, further inquiries can be directed to the corresponding author.

## Ethics Statement

The studies involving human participants were reviewed and approved by the Second Affiliated Hospital of Nanchang University. The patients/participants provided their written informed consent to participate in this study.

## Author Contributions

ZL designed the study, analyzed the data, and wrote the manuscript with contributions from all the authors. YL, HT, and YH collected the clinical data. YH, CH, and ZZ provided the critical comments for this article. All the authors read and approved the final version of the article.

## Conflict of Interest

The authors declare that the research was conducted in the absence of any commercial or financial relationships that could be construed as a potential conflict of interest.

## Publisher's Note

All claims expressed in this article are solely those of the authors and do not necessarily represent those of their affiliated organizations, or those of the publisher, the editors and the reviewers. Any product that may be evaluated in this article, or claim that may be made by its manufacturer, is not guaranteed or endorsed by the publisher.
